# Super-Fast Detection of *Bacillus cereus* by Combining Cellulose Filter Paper-Based DNA Extraction, Multienzyme Isothermal Rapid Amplification, and Lateral Flow Dipstick (MIRA-LFD)

**DOI:** 10.3390/foods14030454

**Published:** 2025-01-30

**Authors:** Shuqiong Yi, Nali Zhou, Yan Ma, Lunzhao Yi, Ying Shang

**Affiliations:** Faculty of Food Science and Engineering, Kunming University of Science and Technology, Kunming 650500, China; yishuqiong20222125011@stu.kust.edu.cn (S.Y.); 20222225040@stu.kust.edu.cn (N.Z.); 20202214055@stu.kust.edu.cn (Y.M.); yilunzhao@kust.edu.cn (L.Y.)

**Keywords:** *Bacillus cereus*, cellulose filter paper, fast DNA extraction, multienzyme isothermal rapid amplification, lateral flow dipstick

## Abstract

*Bacillus cereus* is a widespread foodborne pathogen that can cause food poisoning when present in food at certain levels. Ingesting contaminated food may lead to symptoms such as abdominal pain, diarrhea, and, in severe cases, life-threatening conditions. In this study, a simple and super-fast method for detecting *B. cereus* was developed, which combines cellulose filter paper-based DNA extraction, multienzyme isothermal rapid amplification (MIRA), and lateral flow dipstick (LFD) technology. Initially, PCR was adopted to evaluate the DNA extraction efficiency of the filter paper, followed by the optimization of the lysis formula and extraction conditions. With the above optimization, DNA that can be used for subsequent nucleic acid amplification can be obtained within 3 min. Then, the isothermal amplification of MIRA–LFD was established and optimized to evaluate the detection specificity and sensitivity. Finally, the developed method was applied to detect *B. cereus* in cooked rice samples. The results indicated that the entire amplification procedure of MIRA-LFD only takes 15 min at 39 °C. The whole super-fast detection system could be completed in less than 20 min, from DNA extraction to result interpretation, which achieved a detection limit of 12 fg/μL of DNA concentration, corresponding to approximately 115 CFU/mL in actual samples.

## 1. Introduction

*Bacillus cereus* is a spore-producing Gram-positive bacillus [1], a conditional pathogen capable of causing many diseases [2]. The bacterium is ubiquitous in the natural environment [3] and can be isolated from soil, sediment, water, and different types of food [4,5]. The presence of *B. cereus* has been reported in foods such as infant formula, milk, dairy products, fruits, vegetables, preserves [6,7,8,9], and spices, making it a common foodborne pathogen worldwide [10]. It causes two different types of gastrointestinal disorders: vomiting and diarrhea syndromes. The primary cause of vomiting syndrome is encephalotoxin. The unique emetic encephalotoxin secretion leads to nausea, vomiting, and liver failure [11]. After ingesting *B. cereus*-contaminated foods, the incubation period is typically 0.5–5 h, after which the host experiences vomiting and nausea, with a relatively rapid onset of symptoms. Diarrheal food poisoning is caused by complex enterotoxins [11], such as hemolytic enterotoxin HBL (encoded by *hblA*, *hblC*, and *hblD*), nonhemolytic enterotoxin NHE (encoded by *nheA*, *nheB*, and *nheC*), and cytotoxins (CytK) [12]. In addition to foodborne illnesses such as diarrhea and vomiting, *B. cereus* can lead to a variety of species of diseases, including fulminant bacteremia, meningitis, pneumonia, gas gangrene infection, and endophthalmitis [13]. Therefore, the timely detection of *B. cereus* in food is an effective means to reduce the occurrence of food poisoning. The molecular biological method based on the in vitro amplification of genomic DNA of *B. cereus* is the common detection method at present.

When implementing the detection process, the first step is DNA extraction. At present, the commonly used methods are Cetyl Trimethyl Ammonium Bromide (CTAB) [14,15], Sodium dodecyl sulfate (SDS) [16,17], and commercialized kits [18]. However, these methods have common disadvantages: expensive equipment, high cost, complicated operation process, strict sample pretreatment, and possible false positives. Cellulose filter paper-based DNA extraction has been developed in recent years; however, the exact mechanism of binding cellulose filter paper to DNA is still unclear. Zou et al. [19] found that an untreated cellulose filter paper can quickly collect nucleic acids in seconds and retain them in a single washing step, whereas contaminants present in complicated biological samples may be quickly eliminated. This phenomenon may be due to the paper’s capacity to adsorb DNA. The method uses a cellulose filter paper-based stick with a DNA-binding zone and a zone impervious to water, prepared by dipping half of the cellulose filter paper into molten wax. Given this approach, a series of methods based on the rapid identification of DNA molecules has been established for various biological samples. Cellulose-based DNA binding is ideal for molecular diagnostics as it is inexpensive, portable, disposable, and easily modified [20,21]. This method has been widely used in the detection of various biological samples, such as plant leaves, viruses, and blood [22,23,24]. However, DNA extraction for foodborne pathogens, especially *B. cereus*, has rarely been reported. Thus, DNA extraction has become a rate-limiting step in microbial detection and a limiting factor for rapid detection.

In recent years, isothermal nucleic acid amplification techniques have emerged as a new molecular diagnostic method that can be used as alternative to techniques such as PCR. Several techniques based on isothermal amplification have been developed, such as loop-mediated isothermal amplification (LAMP), recombinase polymerase amplification (RPA), and multienzyme isothermal rapid amplification (MIRA) [25]. MIRA was considered as an improved version of RPA, which belongs to the isothermal amplification technique and can be performed at a constant temperature. At room temperature, the recombinase and primers form a single-stranded nucleotide complex. The complex scans the DNA double-stranded body for homologous sequences. After finding a target region complementary to the primer, the complex invades the double-stranded DNA template using auxiliary proteins and single-stranded binding proteins and forms a D-Loop region at the invasion site. Subsequently, the complex disintegrates, and the polymerase binds to the 3′ end of the primer to initiate chain extension [26]. Depending on the action of the nuclease, the incorporation of specific molecular probes designed in accordance with the template and the use of lateral flow dipstick allows the detection of the final result. MIRA does not require complex instrumentation, and signal amplification can be achieved under simple conditions. This method is characterized by its short reaction time and strong amplification efficiency. Unlike LAMP, PCR, and their derivatives, MIRA also does not depend on complex temperature changes and does not require the design of multiple pairs of primers.

**Scheme 1:**
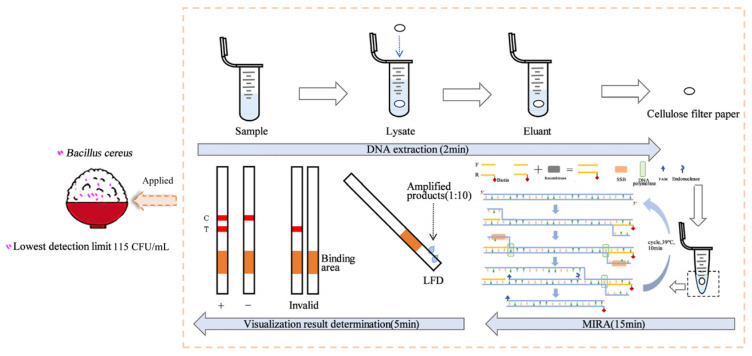
Super-fast detection principle of *Bacillus cereus* by MIRA-LFD.

In this study, cellulose filter paper-based DNA extraction of *B. cereus* was developed and optimized. In order to achieve visual on-site rapid detection without any equipment, multienzyme isothermal rapid amplification (MIRA) was designed, and lateral flow dipstick (LFD) was used to output the detection result (Scheme 1). The whole process of this detection method is simple and super-fast, and can proceed from DNA extraction to detection result output in only 20 min. Similarly, the method does not require large instruments; thus, it is extremely suitable for on-site nucleic acid detection outside the laboratory, which provides technical support for the on-site visual and rapid detection of food safety biogenic risk factors.

## 2. Materials and Methods

### 2.1. Strains, Culture Conditions, and DNA Extraction

Five strains, including *B. cereus* (CMCC(B) 63303), *Escherichia coli* O157 (NCTC 12900), *Salmonella* Typhimurium (CMCC(B) 50094), *Shigella flexneri* (CMCC(B) 51105), and *Listeria monocytogenes* (CMCC(B) 54002), were purchased from Shanghai Luwei Technology Co. (Shanghai, China). All strains were incubated for 24 h in an LB broth medium (Hope Bio-Technology Co., Ltd., Qingdao, China) at 37 °C.

When comparing the method established in this study with commercial kits, and verifying the detection sensitivity of MIRA, the genomic DNA was extracted using Ezup Columnar Bacteria Genomic DNA Extraction Kit (B518255-0100, Shanghai Sangon Biotechnology Co., Ltd., Shanghai, China).

### 2.2. PCR Condition

Nucleic acid amplification was performed by PCR, containing 11.5 μL of 2× SanTaq PCR MIX premix, 1 μL of 10 μmol/L primer each, and 25 μL of ddH_2_O. All primers were synthesized by Shanghai Sangon Biotechnology Co., Ltd. (Shanghai, China). The primer sequences and reaction conditions are shown in Tables S2 and S3. The PCR products were analyzed on 2% agarose gel electrophoresis (containing 10% TS–GelRed, 10 μL TS-GelRed was added to 100 mL 2% agarose).

### 2.3. DNA Extraction of B. cereus Using Cellulose Filter Paper Strip

The prepared LB broth medium was divided into 50 mL triangle bottles; each bottle was divided into 20 mL and sterilized at 121 °C for 20 min. Next, 200 μL of the frozen *B. cereus* liquid was added to the LB broth medium prepared in advance. The membrane was sealed and placed in an incubator at 37 °C for 24 h. Afterward, 1 mL of the activated *B. cereus* sap was placed into a 2 mL centrifuge tube and 500 μL of lysis solution (100 mmol/L pH 8.0 Tris–HCl, 1 mol/L NaCl, and 10 mmol/L EDTA) was added. Then, 100 μL of 0.25 mg/mL chitosan was added to the abovementioned sample, vortexed, and lysed for 5 s. After the lysis of the bacterial solution, the filter paper strip was immersed into a centrifuge tube containing the extraction solution for 5 s to adsorb the DNA and then transferred to a centrifuge tube containing 100 μL of eluent (10 mmol/L pH 8.0 Tris–HCl, 0.1% Tween–20) for 5 s to elute the inhibitors on the filter paper strip that hindered the amplification of downstream nucleic acids. Finally, the filter paper strip was transferred to the amplification system and left for 5 s. This amplification solution was used directly for subsequent amplification.

### 2.4. Condition Optimization of DNA Extraction Based on Cellulose Filter Paper Strip

#### 2.4.1. Production of a Cellulose Filter Paper Strip

**Figure 1:**
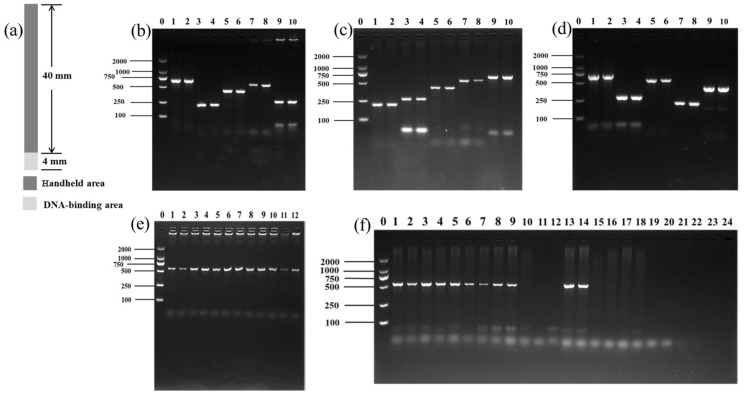
Evaluation of the ability of cellulose filter paper strips to extract bacterial DNA. (**a**) Structural diagram of cellulose filter paper strips. (**b**) Electropherograms of the PCR products extracted from the DNA of five pathogenic bacteria using cellulose filter paper strips. Lane 0, marker; lanes 1 and 2, *E. coli*; lanes 3 and 4, *S. flexneri*; lanes 5 and 6, *S.* Typhimurium; lanes 7 and 8, *B. cereus*; lanes 9 and 10, *L. monocytogenes*. (**c**) Electropherograms of the PCR products extracted from the DNA of five pathogenic bacteria mixed in random proportions. Lane 0, marker; lanes 1 and 2, *S. flexneri*; lanes 3 and 4, *L. monocytogenes*; lanes 5 and 6, *S.* Typhimurium; lanes 7 and 8, *B. cereus*; lanes 9 and 10, *E. coli*. (**d**) Electropherograms of the PCR products extracted from the DNA of five pathogenic bacteria using the kit method. Lane 0, marker; lanes 1 and 2, *E. coli*; lanes 3 and 4, *L. monocytogenes*; lanes 5 and 6, *B. cereus*; lanes 7 and 8, *S. flexneri*; lanes 9 and 10, *S.* Typhimurium. (**e**) Electropherograms of the PCR products of the DNA extracted using six lysates. Lane 0, marker; lanes 1 and 2, lysate 1; lanes 3 and 4, lysate 2; lanes 5 and 6, lysate 3; lanes 7 and 8, lysate 4; lanes 9 and 10, lysate 5; lanes 11 and 12, lysate 6. (**f**) Electropherograms of the PCR products of the DNA extracted by adding chitosan at different concentrations. Lane 0, marker; lanes 1 and 2, control; lanes 3 and 4, 20 μL of 0.25 mg/mL chitosan was added; lanes 5 and 6, 20 μL of 2.5 mg/mL chitosan was added; lanes 7 and 8, 20 μL of 10 mg/mL chitosan was added; lanes 9 and 10, 20 μL of 25 mg/mL chitosan was added; lanes 11 and 12, 20 μL of 50 mg/mL chitosan was added; lanes 13 and 14, 100 μL of 0.25 mg/mL chitosan was added; lanes 15 and 16, 100 μL of 2.5 mg/mL chitosan was added; lanes 17 and 18, 100 μL of 10 mg/mL chitosan was added; lanes 19 and 20, 100 μL of 25 mg/mL chitosan was added; lanes 21 and 22, 100 μL of 50 mg/mL chitosan was added; lanes 23 and 24, empty.

First, a Whatman no.1 cellulose filter paper (Whatman, Little Chalfont, UK) was cut into strips with a size of 2 mm × 20 mm, and a line was drawn with a pencil at 16 mm from the bottom edge of the filter paper, which was then set aside; the sealing film (Shanghai Sangon Biotechnology Co., Ltd., Shanghai, China) was cut into 2 mm × 40 mm and pasted on both sides of the filter paper with a length of 16 mm, forming a handheld area with a length of 40 mm on the filter paper by using the sealing film. The 4 mm area of the filter paper not covered by the sealing film was used as the DNA-binding area. The prepared filter paper strips were sterilized by UV light for 30 min before use and then transferred to a clean glass for use (Figure 1a).

#### 2.4.2. Selection of Lysate Components

Six different lysis buffers were selected to lyse the *B. cereus* bacterial solution. The components of the six lysis buffers and the amount of addition required are listed in Table 1.

In addition, chitosan (Shanghai Sangon Biotechnology Co., Ltd., Shanghai, China) can play a role in protecting DNA from degradation to a certain extent during DNA extraction [31]. After adding 500 μL of lysate to the sample, 20 μL and 100 μL of solutions concentration 0.25 mg/mL, 2.5 mg/mL, 10 mg/mL, 25 mg/mL, and 50 mg/mL chitosan were pipetted and added to the sample solution, and vortexed. No chitosan was used as the control group.

#### 2.4.3. Optimization of DNA Extraction Conditions

In this study, the optimal conditions for the extraction of *B. cereus* DNA from filter paper strips were determined by optimizing the volume of lysate added, the lysis time, the time of immersion of filter paper strips in lysate, the volume of eluent added, and the time of elution of impurities to ensure that the subsequent amplification reaction proceeded smoothly. Therefore, 200 μL, 500 μL, 1 mL, 2 mL, and 5 mL of lysate were added to the samples, and the mixture was vortexed. Different lysis time (5 s, 30 s, 1 min, 5 min, and 10 min), adsorption time of the lysed sample solution to the filter paper strip (5 s, 30 s, 1 min, 5 min, and 10 min), eluent volume (elution of impurities in 100 μL, 500 μL, 1 mL, 2 mL, and 5 mL of the eluent), and elution time of the impurities (5 s, 30 s, 1 min, 5 min, and 10 min) were set, as shown in Table S4 [32,33,34,35,36].

### 2.5. Primer and Probe Designing and Screening for MIRA–LFD

The *nhe* gene of *B. cereus* was downloaded from NCBI (accession number DQ885236.1). The primer and probe were designed using the GenScript primers designer website (https://www.genscript.com/tools/pcr-primers-designer/standard, accessed on 1 April 2024) in accordance with the guidelines of Weifang AmpFuture Biotechnology Co., Ltd. (Weifang, China) and the multiple primer analyzer website to check whether the designed sequences were dimerized or not. The length of the primer is 30–35 bp; the GC content is preferably between 30% and 60%, and the length of the amplification product is 150–300 bp, in which the 5′ end of the downstream primer needs to be labeled with biotin; the length of the probe is 46–52 nucleotides, which should be located between the forward and reverse primers. The 5′ end is modified with carboxyfluorescein (FAM); any base at about 30 nt from the 5′ end is replaced with a dSpacer (tetrahydrofuran [THF]), and the 3′ end is labeled with a C3Spacer-modifying group.

By cross-combining three sets of designed upstream and downstream primers and one probe, nine different sets of primer pairs can be obtained. In the process of primer screening, conduct a negative control test using sterile deionized water in place of the DNA template, omitting DNA completely, to determine if any false positives are observed in the detection results. Nine sets of primers were tested negative, that is, sterile deionized water was used instead of DNA template. The primer–probe combinations with a correct target band size, less miscellaneous bands, and normal negatives were screened. The primer and probe sequences synthesized by Sangon Biotech Co., Ltd. (Shanghai, China) are shown in Table 2.

### 2.6. Conditions Optimization of MIRA–LFD

#### 2.6.1. MIRA–LFD Reaction System

The MIRA amplification reaction system was 50 μL in total, including 29.4 μL of A buffer, 1.0 μL each of forward and reverse primers (10 μmol/L), 0.2 μL of probe (10 μmol/L), 2.0 μL of DNA template, and 13.9 μL of sterile deionized water. The premixed reaction solution was added into a dry powder reaction tube, and 2.5 μL of B buffer was placed in the cap of the tube, mixed upside down, centrifuged immediately, and incubated for 15 min at 39 °C in a metal bath. At the end of the reaction, the amplification product was diluted 10-fold with sterile deionized water and added dropwise onto lateral flow dipsticks. The results were read by observing the quality control line and test line within 5 min. The MIRA–LFD kit was purchased from Weifang AmpFuture Biotechnology Co., Ltd., Weifang, China.

#### 2.6.2. Reaction Conditions Optimization of MIRA–LFD

In this study, the optimal amplification conditions for the MIRA–LFD reaction were determined by optimizing the amplification system, amplification temperature, and amplification time. Different amplification temperatures (33 °C, 36 °C, 39 °C, 42 °C, and 45 °C) and amplification times (5 min, 10 min, 15 min, 20 min, and 25 min) were set, and the combinations of amplification systems are shown in Table 3.

### 2.7. Detection Specificity and Sensitivity of MIRA-LFD

A total of 2 μL of *B. cereus* DNA, *E. coli* DNA, *L. monocytogenes* DNA, *S. flexneri* DNA, and *S.* Typhimurium DNA was added to the MIRA–LFD amplification system solution, and the amplification products were detected. Meanwhile, the specificity of the method was evaluated by using sterile deionized water as the reaction template and negative control.

The *B. cereus* DNA extracted using the kit method was diluted to 1.2 × 10^−1^–1.2 × 10^−7^ ng/μL in a 10-fold gradient, and 2 μL of the extract was taken as the reaction template. In addition, the sensitivity of the method was analyzed by using sterile deionized water as the reaction template and negative control.

### 2.8. Practical Sample Application

Samples were randomly taken from cooked rice in a student cafeteria using a sterile sampling bag. First, 2.5 g of cooked rice was placed into a sterile triangular bottle, combined with 200 μL of *B. cereus* freezing solution as a manually spiked sample, incubated at 37 °C for 24 h, added to 22.5 mL of 0.85% sterile saline, and mixed well. In another sterile triangular vial, 2.5 g of cooked rice was weighed, added to 22.5 mL of sterile saline, and mixed well. This sample served as the blank control. Sterile deionized water was used as the negative control. Bacterial DNA was extracted from 1 mL of manually spiked standard and blank samples using filter paper strips for MIRA–LFD visualization in accordance with the kit instructions. PCR amplification was also performed to verify the consistency of the results. The actual sample sensitivity was analyzed by the 10-fold gradient dilution of manually spiked samples in the range of 10^−1^–10^−6^ CFU/mL. Plates were coated with 1 mL of diluted samples and cultured at 37 °C for 48 h. Two parallels were set up.

## 3. Results

### 3.1. Assessment of DNA Extraction Capability Using Cellulose Filter Paper Strips

In verifying whether a cellulose filter paper can extract bacterial DNA, we used a cellulose filter paper to extract DNA from *B. cereus*, *E. coli*, *S.* Typhimurium, *S. flexneri*, and *L. monocytogenes*, separately. First, we designed the cellulose filter paper as a strip consisting of a handheld end and a DNA-binding end. As shown in Figure 1b, the PCR amplification products of DNA extracted from five foodborne pathogenic bacteria by using filter paper strips were detected by electrophoresis, and all of them showed clear and bright bands at the target bands. As shown in Figure 1c, the PCR amplification products of the template that extracted from the DNA of five pathogenic bacteria mixed in random proportions were detected by electrophoresis, and all of them showed clear and bright bands at the target bands.

The DNA of five pathogenic bacteria was extracted using commercially available kits. As shown in Figure 1d, the amplified products showed clear and bright bands at the target bands. Compared with the filter paper method (Figure 1b), there was no significant difference in the brightness of the two electrophoresis strips. However, DNA extraction using the kit method took 2 h, whereas using the filter paper method only took 2 min.

### 3.2. Selection and Optimization of Lysate Components

In this study, six different lysates were used to extract the DNA of *B. cereus*. As shown in Figure 1e, all six lysates achieved effective amplification, and the electrophoresis of the PCR-amplified products showed the target bands. In addition, the bands were bright and clear, and no non-specific bands were observed. Among them, the electrophoretic bands of PCR amplification products after genomic DNA extraction using no. 2, no. 3, no. 4, and no. 5 lysates were brighter. Based on the preparation components of the six lysates shown in Table 1, the no. 2 lysate has the least composition, and it is the easiest to prepare, which can save costs to a certain extent. Therefore, the no. 2 lysate is the optimal choice.

During the rapid extraction of genomic DNA, the degradation and loss of DNA are a point of concern. Different concentrations of chitosan were added to the original base of no. 2 lysate for the lysis of *B. cereus* to observe whether chitosan had an enhancing effect on DNA extraction. As shown in Figure 1f, in the 20 μL chitosan group, the chitosan concentration of 0.25, 2.5, 10, and 25 mg/mL showed bright bands, and the chitosan concentration of 25 mg/mL showed only one parallel band; in the 100 μL chitosan group, only the chitosan concentration of 0.25 mg/mL showed a strip. Compared with the control group, the brightness of the strip added with 100 μL of 0.25 mg/mL chitosan was stronger, and the parallelism was better than that of the control group. In summary, the DNA extraction effect was remarkably enhanced by adding chitosan to lysate no. 2, and the addition of 100 μL of 0.25 mg/mL chitosan was the optimal condition.

### 3.3. Optimization of DNA Extraction Conditions

**Figure 2:**
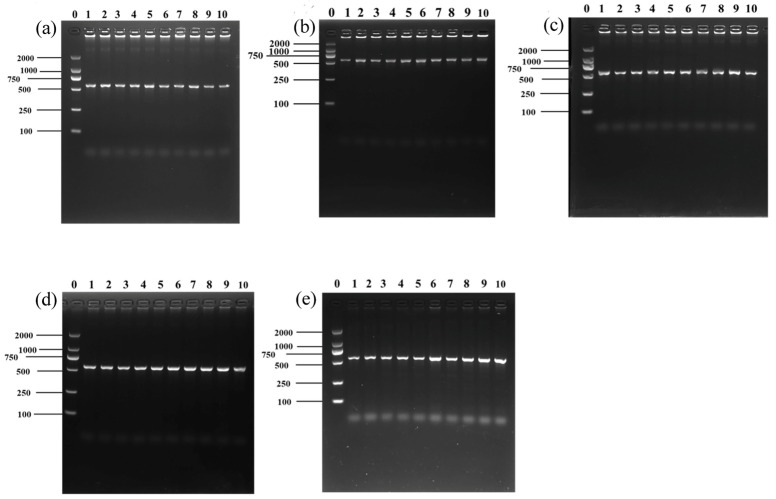
Effects of different extraction conditions on the DNA extracted from *B. cereus*. (**a**) Electropherograms of PCR products of the DNA extracted by adding different volumes of lysate. Lane 0, marker; lanes 1 and 2, 200 μL of lysate was added; lanes 3 and 4, 500 μL of lysate was added; lanes 5 and 6, 1 mL of lysate was added; lanes 7 and 8, 2 mL of lysate was added; lanes 9 and 10, 5 mL of lysate was added. (**b**) Electropherograms of the PCR products of the DNA extracted at different lysing times. Lane 0, marker; lanes 1 and 2, lysis for 5 s; lanes 3 and 4, lysis for 30 s; lanes 5 and 6, lysis for 1 min; lanes 7 and 8, lysis for 5 min; lanes 9 and 10, lysis for 10 min. (**c**) Electropherograms of the PCR products of the DNA extracted using filter paper strips with different times of immersion into the lysate. Lane 0, marker; lanes 1 and 2, filter paper strips immersed in the lysate for 5 s; lanes 3 and 4, filter paper strips immersed in the lysate for 30 s; lanes 5 and 6, filter paper strips immersed in the lysate for 1 min; lanes 7 and 8, filter paper strips immersed in the lysate for 5 min; lanes 9 and 10, filter paper strips immersed in the lysate for 10 min. (**d**) Electropherograms of the PCR products of the DNA extracted with different volumes of eluent. Lane 0, marker; lanes 1 and 2, 100 μL of eluent was added; lanes 3 and 4, 500 μL of eluent was added; lanes 5 and 6, 1 mL of eluent was added; lanes 7 and 8, 2 mL of eluent was added; lanes 9 and 10, 5 mL of eluent was added. (**e**) Electropherograms of the PCR products of the DNA extracted at different impurity elution times. Lane 0, marker; lanes 1 and 2, elution for 5 s; lanes 3 and 4, elution for 30 s; lanes 5 and 6, elution for 1 min; lanes 7 and 8, elution for 5 min; lanes 9 and 10, elution for 10 min.

Based on the abovementioned experimental results, several conditions involved in the DNA extraction process were optimized in this study. First, the volume of lysate added was optimized. As shown in Figure 2a, effective amplification can be achieved in different lysis solution volumes. The electrophoresis bands of PCR amplification products with added 5 mL of lysis solution are weaker than other bands, but the difference is not particularly large. Moreover, no significant difference in the brightness of the remaining bands is found.

Second, the lysis time was optimized, and the results are shown in Figure 2b. During lysis for 5 s, 30 s, 1 min, 5 min, and 10 min, no significant difference in the amount of amplified products was observed, and effective amplification could be achieved.

Third, the immersion time of filter paper strips in the lysate was optimized, and the results are shown in Figure 2c. In addition, no significant difference in the amount of amplified products was observed during adsorption for 5 s, 30 s, 1 min, 5 min, and 10 min. All of the products can achieve effective amplification.

Fourth, the addition volume of the eluent was optimized, and the impurities were eluted in 100 μL, 500 μL, 1 mL, 2 mL, and 5 mL eluents. As shown in Figure 2d, there was no significant difference in the amount of amplification product found in different eluents, and effective amplification can be achieved by all products.

Fifth, the impurity elution time was optimized (Figure 2e). During impurity elution for 5 s, 30 s, 1 min, 5 min, and 10 min, the brightness of the electrophoresis bands of PCR amplification products increased with the increase of elution time.

These five conditions do not greatly influence the DNA extraction of *B. cereus*. Therefore, a lysate volume of 200 μL, lysis time of 5 s, adsorption time of 5 s, elution volume of 100 μL, and elution time of 5 s were selected as the optimal combination in considering the economic cost and time cost.

### 3.4. Primer and Probe Screening for MIRA–LFD

**Figure 3:**
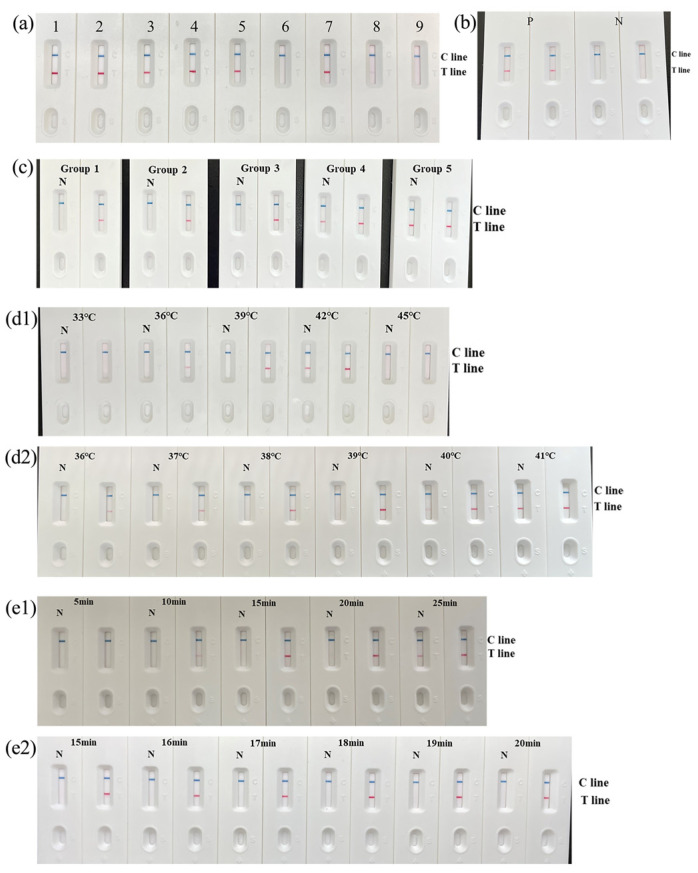
MIRA–LFD detection and optimization of reaction conditions. (**a**) Negative detection of 9 groups of MIRA-LFD primer pairs. (**b**) Cellulose filter paper-based DNA extraction combined with the MIRA–LFD visual detection system was established. (**c**) Screening for optimal amplification system. (**d**) Screening for optimal amplification temperature. (**e**) Screening for optimal amplification time. P, positive control. N, negative control. C line, control line. T line, test line.

The three designed primer pairs and one probe were cross-combined to form nine groups of primer pairs (Table 4). The results of the all-negative test for the nine groups of primer pairs are shown in Figure 3a, with groups 6 and 9 showing no coloration at the test line T and showing blue in control line C, which was negative and normal, and the remaining seven groups showing red at the test line T, which was negative and abnormal, resulting in a false-positive phenomenon. Therefore, the optimal combinations of the screened primer pairs were nhe–F2/R3/P1 and nhe–F3/R3/P1, and the suboptimal combination was nhe–F3/R2/P1. However, the negative control test for group 9 exhibited a noticeable water mark on the test line (T line) initially, and later the water mark of group 9 was no longer visible. In contrast, group 6 appeared very clean, with no color or marks on the T line. Additionally, the control line (C line) of sample 9 was not as bright as that of sample 6, which may interfere with the performance of the lateral flow assay. Subsequently, the primer pairs that yielded false-positive results in the MIRA–LFD reaction were discarded, and a group of 6 primer pairs was selected for subsequent testing. *B. cereus* DNA extracted from filter paper strips was added into the MIRA amplification reaction solution for MIRA–LFD detection. As shown in Figure 3b, the positive group with the addition of a DNA template showed coloration at the test line T, whereas the negative group without the addition of a DNA template did not show coloration at the test line T. The negative group was normal. Therefore, the nhe–F2/R3/P1 combination was selected for subsequent studies.

### 3.5. Optimization of Reaction Conditions for MIRA–LFD

To determine the optimal reaction conditions for MIRA–LFD, the amplification system, amplification temperature, and amplification time were optimized in this study. During the screening of the optimal amplification system solution, five groups of different systems were set up, and the amount of forward and reverse primers and probes added was changed (Table 3). The results are shown in Figure 3c. The systems of 1–3 can achieve effective amplification, and the negatives of the systems of 4 and 5 were colorful at the test line, thereby resulting in false positives. This result indicates that the probability of false positives increases with the increase of probe concentration. Therefore, combination 1 was selected as the best amplification system.

During the screening of optimal amplification temperature, 33 °C, 36 °C, 39 °C, 42 °C, and 45 °C were first set for amplification, and the results of the MIRA–LFD assay are shown in Figure 3(d1). Normal amplification could be achieved at 36 °C–39 °C, and better amplification was achieved at 39 °C. In addition, 33 °C and 45 °C did not produce amplification, whereas 42 °C produced non-specific amplification, indicating negative abnormality. Subsequently, we conducted a more detailed study on the effect of temperature on the MIRA–LFD reaction, and the temperature was set to 36 °C, 37 °C, 38 °C, 39 °C, 41 °C, and 42 °C for amplification. The results of the MIRA–LFD assay are shown in Figure 3(d2), which indicated that amplification could be normal at 36 °C and 39 °C, and the T line was clearer and redder with the increase of temperature. Non-specific amplification occurred at 41 °C and 42 °C. Therefore, 39 °C is the best amplification temperature.

During the screening of optimal amplification time, five groups of amplification time (5 min, 10 min, 15 min, 20 min, and 25 min) were set up. The results of the MIRA–LFD assay are shown in Figure 3(e1). Effective amplification can be achieved at 10 min, 15 min, and 20 min, and no significant difference in the amplification effect was observed after 15 min and 20 min; 5 min did not produce amplification, whereas 25 min produced non-specific amplification. Subsequently, we studied the amplification time in more detail, adjusting the time intervals (15 min, 16 min, 17 min, 18 min, 19 min, and 20 min). As shown in Figure 3(e2), effective amplification could be achieved at each amplification time, and no significant difference in the amplification effect was observed. Therefore, to save detection time, 15 min was selected as the optimal amplification time.

### 3.6. Specificity and Sensitivity of the Complete Method

**Figure 4:**
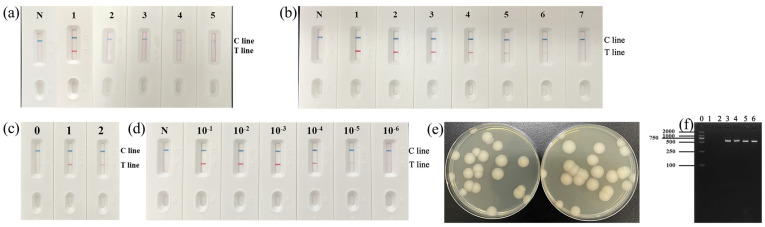
Practical sample application. (**a**) DNA specificity validation. 1, *B. cereus*; 2, *E. coli*; 3, *L. monocytogenes*; 4, *S. flexneri*; 5, *S.* Typhimurium. (**b**) DNA sensitivity analysis: 1, 1.2 ng/μL; 2, 0.12 ng/μL; 3, 12 pg/μL; 4, 1.2 pg/μL; 5, 0.12 pg/μL; 6, 12 fg/μL; 7, 1.2 fg/μL. (**c**) Detection of pathogenic bacteria in cooked rice: 0, blank control; 1, artificially spiked *B. cereus*; 2, artificially spiked mixed pathogenic bacteria. (**d**) Sensitivity analysis. N, negative control. (**e**) Plate count. (**f**) Detection of pathogenic bacteria in cooked rice by using the kit method combined with PCR amplification. Lane 0, marker; lanes 1 and 2, blank control; lanes 3 and 4, artificially spiked *B. cereus*; lanes 5 and 6, artificially spiked mixed pathogenic bacteria. N, negative control; C line, control line; T line, test line.

This study verified the specificity of the method. The results are shown in Figure 4a. The T line of the target strain was colorful, and the test result was positive. On the contrary, the test result of the non-target strain was negative. This result indicates that the method has good specificity and high accuracy.

In the sensitivity analysis of the method, the *B. cereus* DNA used was extracted with the kit. The DNA quality was identified by Nanodrop and its concentration was measured. In addition, the DNA concentration was diluted to 12 ng/μL. The DNA was diluted to 1.2 × 10^−1^–1.2 × 10^−7^ ng/μL in a 10-fold gradient for sensitivity analysis. As shown in Figure 4b, the DNA template at a concentration of 1.2 × 10^−7^ ng/μL did not show coloration at the T line, and the test result was negative. By contrast, the DNA template at 1.2 × 10^−1^–1.2 × 10^−6^ ng/μL showed coloration at the T line, and the test result was positive. Therefore, the method can detect a minimum DNA concentration of 12 fg/μL.

### 3.7. Practical Sample Application

In this study, actual sample contamination was simulated by artificially adding *B. cereus* to one portion of 2.5 g of cooked rice and background interfering bacteria to another portion of 2.5 g of cooked rice. Cooked rice without any added bacteria was used as a blank control. As shown in Figure 4c, the samples spiked with *B. cereus* and mixed bacteria showed coloration at the T line, and the test results were positive. Moreover, the results of the blank control test were negative. The sensitivity of the sample spiked with *B. cereus* was analyzed by 10-fold gradient dilution. As shown in Figure 4d, the T line of the first six dilution gradients showed coloration, and the T line of the seventh dilution gradient did not show coloration. Furthermore, the test results were negative. The result of plate counting was 115 CFU/mL (Figure 4e).

The kit method combined with PCR amplification was used to detect the electrophoresis of three samples. As shown in Figure 4f, the target bands were displayed in both samples with artificially added bacteria, and no bands were displayed in the blank samples. The detection results were consistent with those of the MIRA–LFD filter paper strip method.

## 4. Discussion

Foodborne illness outbreaks caused by *B. cereus* are common worldwide and pose a threat to public health. As reported, the contamination rate of *B. cereus* in food samples was as follows: cereals, 67.6%; pastry products, 46.2%; cooked food, 40.8%; cooked poultry meat, 32.7%; seafood products, 32.3%; spices, 28.8%; canned products, 16.7%; raw poultry meat, 9.4%; fresh-cut vegetables, 5.0%; and dairy products, 4.8% [37]. Many countries and regions have established legal regulations that require monitoring and controlling the presence of *B cereus* in food to ensure that it meets certain safety standards, thereby protecting consumer health. Therefore, it is essential to monitor *B. cereus* in food safety testing to reduce the risk of food contamination and safeguard the health and safety of consumers.

New molecular technologies for point-of-care testing are developing at an unprecedented rate. Nucleic acid-based molecular tests are characterized by high sensitivity, specificity, and speed [38]. However, a major bottleneck in molecular diagnostics is their reliance on nucleic acid extraction and purification, as well as a relatively time-consuming and laborious process that is difficult to perform outside of modern laboratory environments (such as field testing). Hammouda et al. [39] designed a pipette gun tip device containing a cellulose filter paper, extracted DNA from the embryos or fins of transgenic *Oryziaslatipes* and *Daniorerio*, and rapidly classified different gene-edited types of transgenic fish through PCR amplification. In addition, many studies have been conducted on the use of a cellulose filter paper to extract DNA from biological samples for rapid detection, such as the detection of rice infected with *Magnaporthe oryzae*, tomato infected with *Cucumber mosaic virus*, and the rapid assessment of whether the total number of colonies of *Campylobacter* parasitic in chickens is higher than the industry standard [40,41,42].

To make the method suitable for on-site detection, the present study combined filter paper extraction, MIRA, and LFD to shorten the detection time to 20 min. Moreover, the method can extract *B. cereus* DNA directly from cooked rice, which is convenient and rapid compared with the expensive and slow traditional detection methods in the laboratory.

In this study, nucleic acid extraction and isothermal amplification were key. However, the cellulose filter paper can absorb a limited amount of sample, approximately 25% of the total sample volume, which resulted in a low amount of nucleic acid that could be retained on the filter paper in the subsequent steps, leading to the low detection sensitivity of the subsequent nucleic acid amplification method. Therefore, based on the cellular structure of the samples and the information reviewed, corresponding changes were made in the production of filter papers, the components of the lysate, and the lysis conditions to achieve the optimal conditions that would allow for the extraction of a good quality and quantity of *B. cereus* DNA. In the fabrication of filter paper strips, the purchased enzyme-free PET transparent film was cut into appropriate sizes and affixed to both sides of the strips to form the handheld end. The design of the filter strip eliminates the tedious step of moving the filter sheet to the next centrifuge tube by using a tool (such as a disposable tip). The operation time is saved, and the addition of a PET film does not weaken the extraction effect of DNA. During DNA extraction, the degradation of DNA is inevitable, and its degradation can only be reduced to a certain extent. Therefore, 100 μL of 0.25% chitosan solution was added during sample lysis. Chitosan is the most important derivative of chitin and the second largest natural polymer in the world after cellulose. Chitosan and its derivatives have been characterized as safe and efficient cationic carriers with high biodegradability and biocompatibility, non-toxicity, and low cost [43]. In recent years, chitosan has also been widely used and studied for its ability to protect DNA from nuclease degradation and to transfer DNA to various cells [31]. In this study, we found that the amount of lysate and the length of lysis time did not have a great effect on the DNA extraction effect of *B. cereus*. Therefore, 200 μL of lysate, 5 s of lysis time, 5 s of adsorption time, 100 μL of eluents, and 5 s of elution time were selected as the optimal conditions to save economic and time costs.

In this study, MIRA was combined with LFD to achieve visual detection of *B. cereus*. We also designed and screened the most suitable amplification primers and optimized the amplification temperature, amplification time, and amplification system. The amount of primers added also affects amplification; when a small amount of primers are added, the quantity of product decreases, which can easily lead to false negatives, whereas adding a large amount of primers leads to the synthesis of non-specific products, resulting in false positives [44]. Therefore, we consider that the amplification system is the most important influencing factor. We found that MIRA–LFD has good amplification at 36 °C–40 °C, and neither too high nor too low a temperature could achieve effective amplification. In addition, amplification does not occur within 5 min; it occurs around 10 min. Moreover, amplification is more pronounced within 20 min as the amplification time extends. To shorten the whole assay time and ensure the efficiency and sensitivity of the assay, the amplification time of MIRA–LFD was 15 min. We further verified the specificity and sensitivity of MIRA-LFD. The T line of the added DNA of the target strain was colored, and the test result was positive; the non-target strain was detected as negative. The lowest detectable DNA concentration was 12 fg/μL. This result indicates that the method has good specificity and high sensitivity.

Finally, we performed an actual sample spiking experiment. The results indicated that *B. cereus* could be successfully detected in cooked rice spiked with target strains and mixed bacteria. The established cellulose filter paper-based DNA extraction combined with MIRA–LFD visualization showed sensitivity up to 115 CFU/mL. DNA was extracted from cooked rice using the kit method and amplified by PCR. The amplified products were verified by electrophoretic detection. The detection results of the samples spiked with target strains and mixed bacteria were consistent with those of MIRA–LFD. Overall, this study is a rapid assay that can be completed in less than 20 min from DNA extraction to result determination. By contrast, LAMP and real-time fluorescence quantitative PCR (qPCR) usually take about 1 h to complete, and they do not include DNA extraction [45]. However, due to MIRA’s high sensitivity, the issue of nucleic acid contamination becomes quite serious. This contamination arises from aerosols during nucleic acid extraction or from exposure when opening the lid after amplification. Therefore, it is essential to develop a fully sealed detection device to prevent DNA contamination in the further study.

## 5. Conclusions

In this study, cellulose filter paper-based DNA extraction in combination with MIRA–LFD was used for visual detection of *B. cereus*. The detection can be completed in less than 20 min from DNA extraction to result determination. This study combined DNA extraction and rapid amplification, and the results could be observed by the naked eye without using any large-scale instrument, which is easy to operate, rapid, and consistent with the field testing outside of the laboratory. However, there are still some potential limitations to this study; only one MIRA response and one LFD assay could be completed at a time in this study. Moreover, in MIRA amplification, obtaining multiple target sequences in one reaction requires more in-depth studies by a wide range of researchers.

## Figures and Tables

**Table 1:** Six different pyrolysis liquid components and the required amount of addition.

No.	Lysate Components	Lysate Volume	Eluent Volume	Reference
1	1.5 M Guanidine Isothiocyanate, 50 mM Tris • HCl (pH 8.0), 20 mM EDTA, 1% Tween–20, 2 mg/mL DTT, 40 mg/mL TritonX-100, 100 mM NaCl.	500 μL	200 μL	[22]
2	100 mM Tris•HCl (pH8.0), 1 M NaCl, 10 mM EDTA	200 μL	100 μL	[27]
3	50 mM Tris•HCl (pH8.0), 150 mM NaCl, 2% PVP, 1% Tween–20	50 μL	200 μL	[19]
4	20 mM Tris•HCl (pH8.0), 25 mM NaCl, 2.5 mM EDTA, 0.05% SDS	500 μL	1750 μL	[28]
5	800 mM Guanidine Hydrochloride, 50 mM Tris • HCl (pH 8.0), 0.5% Triton–100, 1% Tween–20	100 μL	500 μL	[29]
6	2% NaCl, 2% EDTA, 0.2% Glycerol, 0.2% Polyethylene Glycol, 0.2% Tween–80, 0.01% PVP–K30	500 μL	500 μL	[30]

**Table 2:** MIRA–LFD primer and probe sequences.

Name	Sequence (5′→3′)
nhe–F1	agtaggtggaggtacaacgggaattgttttag
nhe–F2	aatgcacttattattggttcctctgtagccac
nhe–F3	aggcccaattgcgattataggtggtacagttg
nhe–R1	Biotin–aacgctgtaatcgcagtgtcaattgtatttgt
nhe–R2	Biotin–cacaagctcttcaggactaatagaatctacat
nhe–R3	Biotin–gctatcttttgcgatgctaagatcttctttca
nhe–P1	[FAM]–aataacaggtaaaattactactgcacaatt[THF]gaagtagcagggtta–[C3spacer]

**Table 3:** MIRA–LFD amplification system combinations.

Name	Group 1	Group 2	Group 3	Group 4	Group 5
A buffer	29.4 μL				
B buffer	2.5 μL				
Forward primer (μL)	1	1	2	3	5
Reverse Primer (μL)	1	1	2	3	5
Probe (μL)	0.2	0.3	0.6	0.9	1.5
DNA	2 μL				
Water (μL)	13.9	13.8	11.5	9.2	4.6
All	50 μL				

**Table 4:** MIRA–LFD primer cross combination.

No.	Primers			Result
1	nhe–F1	nhe–R1	nhe–P1	+
2		nhe–R2		+
3		nhe–R3		+
4	nhe–F2	nhe–R1		+
5		nhe–R2		+
6		nhe–R3		–
7	nhe–F3	nhe–R1		+
8		nhe–R2		Delicacy +
9		nhe–R3		–

## Data Availability

The original contributions presented in this study are included in the article/Supplementary Materials. Further inquiries can be directed to the corresponding author.

## Supplementary Materials

The following supporting information can be downloaded at: https://www.mdpi.com/article/10.3390/foods14030454/s1, Table S1. Reagents and equipment used in this experiment; Table S2. PCR primer sequences for five foodborne pathogens; Table S3. PCR amplification reaction conditions for five foodborne pathogens; Table S4. Optimization of DNA extraction conditions.
