# Supplementary material for: Super-Fast Detection of Bacillus cereus by Combining Cellulose Filter Paper-Based DNA Extraction, Multienzyme Isothermal Rapid Amplification, and Lateral Flow Dipstick (MIRA-LFD)
Source: Foods. 2025 Jan 30;14(3):454. doi: 10.3390/foods14030454 (PMC11817700; doi:10.3390/foods14030454)
Supplement: Supplementary file 1 [file foods-14-00454-s001.zip › foods-3440049-supplementary.pdf]

Table S1. Reagents and equipment used in this experiment.

Name	Manufacturer	Location
Agar	Shanghai Sangon Biotechnology Co., Ltd	Shanghai, China
Agarose	Shanghai Beijing Biotechnology Co., Ltd	Shanghai, China
DL2000 Marker	Beijing Qingke Biotechnology Co., Ltd. Kunming Branch	Beijing, China
2×SanTaq PCR MIX premix	Shanghai Sangon Biotechnology Co., Ltd	Shanghai,China
TS-GelRed	Beijing Qingke Biotechnology Co., Ltd. Kunming Branch	Beijing, China
Guanidine Isothiocyanate	Shanghai Macklin Biochemical Co., Ltd	Shanghai Macklin Biochemical Co.,Ltd.
Tris (hydroxymethyl) methyl aminomethane (Tris)	Shanghai Sangon Biotechnology Co., Ltd	Shanghai, China
NaCl	Tianjin Fengchuan Chemical Reagent Technology Co., Ltd	Tianjin, China
Ethylenediaminetetraacetic acid (EDTA)	Shanghai Chemical Reagent Co., Ltd	Shanghai, China
Tween–20	Shanghai Sangon Biotechnology Co., Ltd	Shanghai, China
DL-Dithiothreitol (DTT)	Shanghai Sangon Biotechnology Co., Ltd	Shanghai, China
Triton-100	Shanghai Sangon Biotechnology Co., Ltd	Shanghai, China
Polyvinylpyrrolidone (PVP)	Xilong Chemical Co., Ltd	Shanghai, China
Polyvinylpyrrolidone K30 (PVP-K30)	Shanghai Macklin Biochemical Co., Ltd	Shanghai, China
Guanidine Hydrochloride	Shanghai Macklin Biochemical Co., Ltd	Shanghai, China
Glycerin	Tianjin Fengchuan Chemical Reagent Technology Co., Ltd	Tianjin, China
Polyethylene Glycol	Shanghai Macklin Biochemical Co., Ltd	Shanghai, China
Tween–80	Shanghai Macklin Biochemical Co., Ltd	Shanghai, China
Sodium dodecyl sulfate (SDS)	Shanghai Macklin Biochemical Co., Ltd	Shanghai, China
HCl	Tianjin Fengchuan Chemical Reagent Technology Co., Ltd	Tianjin, China
LDZF-75KB Vertical autoclave	Shanghai Shen'an Medical Device Factory	Shanghai, China
SW-CJ-2FD Double-person single-sided purification workbench	Shanghai Hujing Medical Equipment Co., Ltd	Shanghai, China
DHP-9051 Microbial incubators	Shanghai Yiheng Scientific Instrument Co., Ltd	Shanghai, China
ABI SimpliAmp PCR thermal cycler	Thermo Fisher Scientific	USA
Microplate thermostatic shaker WKB-100	Tuohe Electromechanical Technology (Shanghai) Co., Ltd	Shanghai, China

Table S2. PCR primer sequences for five foodborne pathogens.

Name	sequence	lengths	References
Escherichia coli O157	ECO157F：ATCGTGAACTGGGAGCTAAAG<br>ECO157R：CTCCCATGTCTCCAAATACT	664pb	[32]
Salmonella Typhimurium	TtrC-F：ACTGCCGATAAATGCACGTT<br>TtrC-R：CTTTTTTCCGCCAGTGAAGA	418pb	[33]
Shigella flexneri	IpaHIF：CGAAATTCTGGAGGACATTG<br>IpaHIR：TCATTCTCTTCACGGCTTC	210pb	[34]
Listeria monocytogenes	LM-F：GATACAGAAACATCGGTTGGC<br>LM-R：GTGTAATCTTGATGCCATCAG	274pb	[35]
Bacillus cereus	nheC-F：GCGGATATTGTAAAGAATCAAAATGAGGT<br>nheC-R：TTTCCAGCTATCTTTCGCTGTATGTAAAT	557pb	[36]

Table S3. PCR amplification reaction conditions for five foodborne pathogens.

Name	reaction conditions	References
Escherichia coli O157	pre-denaturation at 95 °C for 5 min, 95 °C for 45 s, 52 °C for 30 s, 72 °C for 45 s, 35 cycles, extension at 72 °C for 10 min	[32]
Salmonella Typhimurium	pre-denaturation at 95 °C for 5 min, 95 °C for 30 s, 58 °C for 30 s, 72 °C for 30 s, 30 cycles, extension at 72 °C for 10 min	[33]
Shigella flexneri	pre-denaturation at 94 °C for 5 min, 94 °C for 30 s, 52.6 °C for 30 s, 72 °C for 30 s, 30 cycles, extension at 72 °C for 7 min	[34]
Listeria monocytogenes	pre-denaturation at 95 °C for 5 min, 95 °C for 60 s, 59 °C for 60 s, 72 °C for 60 s, 35 cycles, extension at 72 °C for 5 min	[35]
Bacillus cereus	pre-denaturation at 95 °C for 5 min, 95 °C for 30 s, 55 °C for 30 s, 72 °C for 30 s, 30 cycles, extension at 72 °C for 10 min	[36]

Table S4. Optimization of DNA extraction conditions

Name	variable					
volume of lysate	-	200 μL	500 μL	1 mL	2 mL	5 mL
lysis time	-	5s	30s	1 min	5 min	10 min
adsorption time	-	5s	30s	1 min	5 min	10 min
volume of eluent	-	100 μL	500 μL	1 mL	2 mL	5 mL
eluent time		5s	30s	1 min	5 min	10 min
